# Atopic Dermatitis in Domestic Animals: What Our Current Understanding Is and How This Applies to Clinical Practice

**DOI:** 10.3390/vetsci8070124

**Published:** 2021-07-02

**Authors:** Rosanna Marsella

**Affiliations:** Department of Small Animal Clinical Sciences, College of Veterinary Medicine, University of Florida, 2015 SW 16th Avenue, Gainesville, FL 32610, USA; Marsella@ufl.edu

**Keywords:** atopic syndrome, dermatitis, dogs, cats, horses

## Abstract

Atopic dermatitis is a clinical syndrome that affects both people and animals. Dogs closely mimic the complexity of the human skin disease, and much progress has been made in recent years in terms of our understanding of the role of skin impairment and the identification of new treatments. Cats and horses also develop atopic syndromes which include both cutaneous and respiratory signs, yet studies in these species are lagging. It is now recognized that atopic dermatitis is not a single disease but a multifaceted clinical syndrome with different pathways in various subgroups of patients. Appreciating this complexity is clinically relevant as we develop more targeted treatments which may work well in some patients but not in others. Different phenotypes of atopic dermatitis have been described in dogs, and it is possible that phenotypes related to breed and age may exist in other animals similar to how they are described in people. The awareness of different mechanisms of disease leads to the desire to correlate different phenotypes with specific biomarkers and responses to treatment. In this review, the current understanding and updated information on atopic syndrome in animals are described, highlighting opportunities for further studies in the future.

## 1. Introduction

Atopic dermatitis is one of the manifestations of atopic disease. In people, dermatitis is typically the first manifestation of atopic disease and can be followed by respiratory disease later in life as part of what is called the “atopic march” [1]. Atopic dermatitis affects people and animals and, in some species (e.g., dogs), is the most prevalent manifestation of atopic disease. Atopic dermatitis in dogs has become increasingly common as exposure to indoor environments and processed foods has increased in our pets. Canine atopic dermatitis has characteristics, both clinically and immunologically, that are strikingly similar to the human counterpart [2,3]. In dogs, progression to respiratory signs has been described in colonies of atopic dogs [4], but it does not seem to be a common observation in clinical practice.

Much progress has been made in our understanding of canine atopic dermatitis in recent years [5]. The availability of colonies of atopic research dogs has greatly helped to shed light on the complex pathogenesis of this condition. In research settings, controlled studies involving allergen challenges can be done to observe the development of lesions and better understand the dynamic changes of inflammatory cytokines over time. In these settings, multiple biopsies can be taken, and the measurement of various inflammatory mediators at various time points is possible [6,7]. The availability of these models has been instrumental in identifying new potential targets, such as IL-31 [8]. The identification of new targets has led to the development of new drugs currently available in clinics to provide relief to animals that spontaneously develop this frustrating and chronic disease.

## 2. Dogs

### 2.1. Evolution of Our Understanding

Our comprehension of canine atopic dermatitis has greatly improved in the last decade [9]. The development of the clinical disease is the result of a complex interaction between genetic and environmental factors. While, in the past, IgE was considered the most important player in the pathogenesis, and much of the emphasis was placed on mast cells and histamine, it is now accepted that IgE may be an epiphenomenon. Many other factors besides IgE and histamine are now known to play a role in this complex [10]. Recent research has focused on the role of the skin barrier both in terms of ultrastructural alterations and dysbiosis [11] and the role of various lymphocytic populations [12,13,14]. This evolution in our understanding has been reflected in how clinical cases are managed. In the past, antihistamines were widely advocated for treatment and prevention of flares. Currently, the emphasis on the use of antihistamines has decreased [15], as controlled studies have failed to show the beneficial effect of antihistamines in a double-blind, placebo-controlled fashion [16]. Despite this, antihistamines may still be prescribed in practice [17] as part of a multimodal approach.

As part of the newer approach, increased attention has been given to the restoration of the skin barrier through the application of sphingolipid and fatty acid emulsions [18,19,20]. This approach has been shown to restore some of the abnormalities of the atopic skin and have a positive effect on clinical signs. Topical therapy has also gained much emphasis for the treatment of secondary bacterial infections [21,22] due to the increase in antimicrobial resistance. Thus, while in the past, the use of topical antimicrobial products was considered an adjunctive therapy, now it is frequently advocated as a monotherapy whenever possible to minimize the use of systemic antibiotics.

### 2.2. Clinical Features, Allergy Tests, and How to Make A Diagnosis

**Figure 1:**
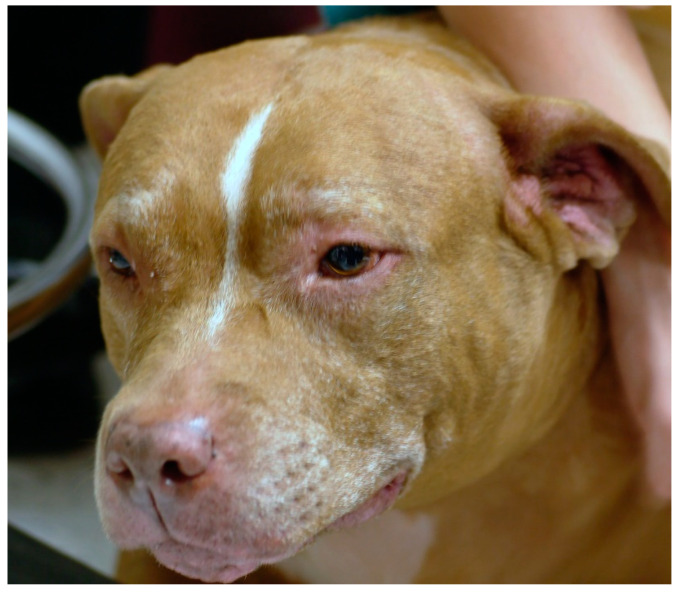
The muzzle, periocular area, and ears are predilected sites for atopic dermatitis in dogs, as shown in this patient.

Atopic dermatitis in dogs manifests as a pruritic inflammatory disease that affects body areas where the allergen is more readily absorbed epicutaneously. Examples of these areas are folds and areas with thinner skin and less hair. Examples include the antebrachial region, the axillae, and the inguinal area. The muzzle, periocular region, pinnae, and interdigital areas are other contact areas where exposure to the allergen is common. Thus, atopic dermatitis has characteristic predilected sites (Figure 1).

Each patient has some characteristic body areas that are prone to clinical flares. For example, some dogs present with inflammatory otitis externa, while others show their disease as pododermatitis. Recurrent otitis externa can be, for some patients, the only sign of atopic dermatitis for a while. Thus, it is important to control the underlying allergic process when patients develop secondary bacterial and yeast otitis on a seasonal basis.

It is important to realize, however, that the body sites affected by atopic dermatitis are not pathognomonic for this disease. For example, the face and feet can be affected by many other conditions, such as demodicosis or contact allergy. Thus, it is important to consider and rule out other differential diagnoses before making a clinical diagnosis of atopic dermatitis. We still lack a diagnostic test for atopic dermatitis. The diagnosis of atopic dermatitis is clinical and based on compatible history, clinical signs, and exclusion of other pruritic diseases. While we still rely on serology testing and intradermal testing to identify possible offending allergens to include in immunotherapy, it is understood that neither of these “tests” can be used for diagnostic purposes. Instead, “allergy tests” should be used *after* a clinical diagnosis of atopic dermatitis has been made [23] with the main intent to formulate allergen-specific immunotherapy. This is an important concept in practice as these tests cannot be used to discriminate between an itchy dog due to atopic disease and an itchy dog affected by parasites. When considering serology tests, it is also important to note that the presence of IgEs against cross-reactive carbohydrate determinants has been documented in dogs. These IgEs are clinically irrelevant but can lead to many false positive results when using serology tests. Currently, some companies treat serum samples to block these IgEs to decrease false positive results and improve the accuracy of their serology test [24].

### 2.3. Identification of Triggers

While foods can be trigger factors for atopic dermatitis in dogs, as is the case in children, we still tend to separate a food-induced disease from atopic dermatitis. Typically, the term atopic dermatitis is used to refer to a pruritic skin disease in which food and fleas have been ruled out, and we are left with a diagnosis by exclusion of “environmentally induced allergic dermatitis”. Thus, although foods are potential triggers of atopic dermatitis-type clinical signs, most clinicians still tend to use the term atopic dermatitis as equivalent to environmental allergies. The reality is that atopic dogs in which environmental triggers are important may be clinically indistinguishable to some in which foods are the trigger [25,26]. As avoidance of triggers is important in managing clinical flares, the identification of a possible role of foods in affecting the severity of the disease is a critical component of the management of nonseasonal atopic dogs. Importantly, there are some patients that look clinically indistinguishable from our classic atopic dogs but for whom environmental triggers cannot be identified, at least with the current tests available for use. For that subset of patients, we tend to use the terms “intrinsic atopic dermatitis or atopic-like disease” [27]. These cases represent an additional challenge as we are not able to use allergen-specific immunotherapy, and we are limited in only considering symptomatic therapies. Based on the current evidence in those cases, it appears that the clinical characteristics of those dogs are similar to the “extrinsic” cases in which an environmental trigger can be identified. The response to treatments also appears to be the same, although we only have very few studies with a low number of cases. This is an area in which we need to further examine this subpopulation to see if they constitute an early stage of the more classic atopic dermatitis or a specific subset. In other words, it is possible that these patients have not had sufficient time to develop an allergic response, and if they were tested later in life, they may be positive in our traditional tests. It may also be that these patients do not have skin barrier impairments, and that is a protective factor against the development of an epicutaneous sensitization toward environmental allergens.

### 2.4. The Role of Skin Barrier in Canine Atopic Dermatitis

What we have learned in recent years is how important the skin barrier is for atopic dermatitis [28] and the propensity to epicutaneous sensitization to allergens [29]. Indeed, the epicutaneous route of exposure is important for both sensitization [30,31] and for elicitation of clinical signs [31,32]. While we are still lacking definitive evidence of a primary skin barrier impairment in atopic dogs, we do know that secondary skin barrier damage exists, as inflammation and self-trauma deteriorate the barrier function of the skin [33]. A damaged skin is more prone to absorb what it encounters and is prone to develop an allergic response to allergens. This is because damaged keratinocytes release cytokines such as thymic stromal lymphopoietin (TSLP), which promotes a T helper 2 response and the development of an allergic response. Thus, frequent removal of allergens from the skin of allergic dogs is crucial to minimize exposure and the worsening of inflammation. We know that the skin of atopic dogs is more alkaline and that it loses more water than normal skin. While in people, atopic skin is accepted to be drier than normal skin, the skin of atopic dogs does not appear to have decreased hydration, at least based on the current studies we have so far [34]. It is possible that our current methodologies are not sensitive enough or that despite the increased loss of water in atopic dogs, the hydration is still within normal limits. This could be linked to the fact that atopic dogs have been reported to have an increased gene expression of proteins such as filaggrin [35,36], whose breakdown products (natural moisturizing factors) are important for hydration.

As the interest in the skin barrier and keratinization has increased in the past decade, several studies have investigated the role of filaggrin in canine atopic dermatitis. Filaggrin mutations have been reported as one of the most documented risk factors for atopic dermatitis in people [37,38]; thus, it was natural to address this issue in dogs. We now know that more than one filaggrin-type protein exists in dogs, although the exact function of filaggrin 2 in dogs is not clear [39]. Both filaggrin-type proteins are expressed in the upper layers of the skin and play a role in the differentiation of the epidermis and in the hydration of the skin. While in people, filaggrin loss-of-function mutations have been identified as major risk factors for the development of atopic dermatitis, this does not appear to be case in most breeds of dogs [40]. It is possible that in the future we will discover other players that may be more relevant for dogs. Research toward establishing a correlation between the severity of clinical signs and gene expression in the skin of atopic dogs has shown that genes relevant to skin barrier formation and immune function were altered [41]. Most of the studies on the genetics of canine atopic dermatitis had small sample sizes, which hindered their ability to detect factors given the heterogeneity of this condition and the variation of breeds [42].

When challenged with an allergen, atopic dogs attempt to compensate for the insult with an increased production of filaggrin, and an increased expression of enzymes responsible for filaggrin degradation has been described in experimental models of canine atopic dermatitis [43]. The increased proteolytic activity of atopic skin has been reported in atopic people, and it is linked to the increased pH [44]. Clinically speaking, it may be important to acidify atopic skin to both decrease the proliferation of bacteria and yeasts and to decrease the proteolytic activity of ceramidases and proteases that degrade lipids in the skin and increase the rate of desquamation.

### 2.5. Factors Playing A Role in the Development of Clinical Disease

As mentioned previously, the actual development of clinical signs is the result of a combination of genetic and environmental factors [45]. Many genes have been considered as candidates for canine atopic dermatitis [46]. The increased prevalence of atopic dermatitis may not only be linked to a preferential breeding of atopic individuals but also to a change in environmental conditions. In people, according to the “hygiene theory”, it is documented that decreased exposure to parasites and beneficial bacteria predisposes one to the development of atopic dermatitis [47,48]. A similar situation may apply to our pets. While pets in the past were more exposed to an outdoor environment with less exposure to house dust mites and more exposure to parasites and bacteria, the current conditions of ingesting processed foods rather than raw diets, an increased exposure to indoor environments and house dust mites, and a decreased exposure to beneficial bacteria may contribute to an increased development of clinical signs of atopic dermatitis [49,50].

**Figure 2:**
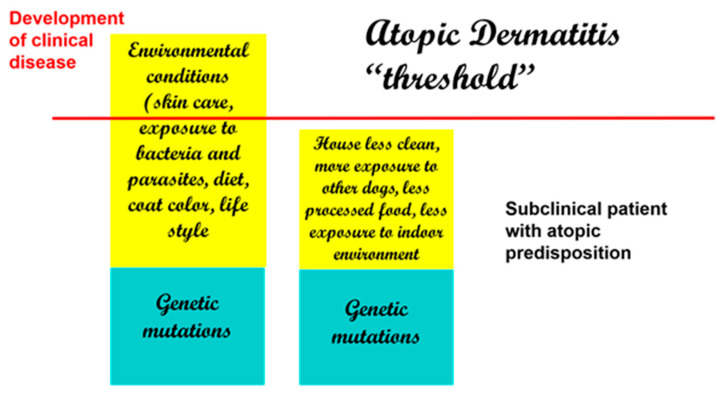
Development of clinical disease is the result of genetic and environmental factors. Decreased exposure to beneficial bacteria and increased exposure to indoor allergens and processed foods are some of the reported risk factors in dogs.

If there were an actual “threshold for development of atopic dermatitis” (Figure 2), we can assume that each factor is additive (from genetic factors to environmental factors) and that once the threshold is achieved, clinical disease ensues. Thus, when genetic factors are considered, it is possible that more than one may be necessary to lead to disease development.

Therefore, with genetic factors being considered a constant, changes in environmental conditions can, by themselves, determine an increased development of clinical disease. This is supported by some studies in the veterinary literature that have linked the development of atopic dermatitis in dogs to dietary habits, lifestyle, and living conditions [51,52]. Of interest, coat color has been linked to the development of atopic dermatitis, and having more than 50% of a white coat color has been reported to be a risk factor [51].

### 2.6. Phenotypes

The concept of phenotypes of atopic dermatitis has been investigated in human medicine for a long time. The term phenotype is intended to emphasize the interaction between genetics and environmental factors. Identification of phenotypes is important as we progress to a more personalized approach to treatment [53,54]. As atopic dermatitis is a heterogenous disease, identification of subgroups of patients is important for the sake of treatment success. Phenotypes of atopic dermatitis in people have been described based on age [55] and ethnicity [56,57,58]. In people, different subgroups appear to have peculiar pathways and key cytokines that play a role, thus requiring different treatments. In dogs, our knowledge of phenotypes is limited. Clinical phenotypes of canine atopic dermatitis have been described based on breeds and distribution of lesions, but no link has been drawn to specific markers and responses to treatment [59].

### 2.7. Strategies for Treatment and Options Available for Atopic Dogs

New research has also focused on the identification of new targets for treatment to minimize the use of broad-spectrum therapies, such as glucocorticoids and cyclosporine, and to use more targeted approaches, such as biologics targeting key cytokines. In this respect, IL-31 has received much attention for its role in canine atopic dermatitis [60]. IL-31 is produced by TH2 cells, and many cells ranging from immune cells to keratinocytes and nerve fibers have receptors for this cytokine [61]. Its role in the mediation of pruritus has gained attention [62], but it important to emphasize that IL-31 also modulates keratinocyte proliferation and differentiation [63]. Strategies to target IL-31 in dogs have ranged from the use of a caninized monoclonal antibody [64] to vaccinating dogs against their own IL-31 [65], although this latter approach is currently only experimental. As atopic dermatitis is a syndrome with different pathomechanisms, not all dogs treated with a biologic aimed at targeting this cytokine respond. This is something to be expected as we move toward more targeted therapies. Nevertheless, this approach is revolutionary in veterinary medicine as biologics offer the freedom of not having to worry about drug interactions and can be considered for patients with a prior history of demodicosis or neoplasia, where other treatments may not be ideal.

Of major importance, we are now appreciating the value of a “proactive approach” when managing cases rather than a “reactive approach” [66]. As these dogs are very likely to flare at some point, it is important to do what is possible to prevent the flares rather than to wait for them to occur and then start treatment. This can be done with topical therapy in areas prone to flaring to minimize the need of rescue medications [67]. If we wait for the flares to occur, we may need more medication and of a larger spectrum, while the proactive approach can now be used with more targeted treatments such as lokivetmab [68]. Over time, fewer flares and fewer medications are needed to make the patient comfortable compared to the philosophy of waiting until the animal flares and then starting the treatment.

Allergen-specific immunotherapy is still the only approach that may potentially alter the course of the disease and minimize future sensitizations. Different routes of administration have been reported in the literature, with the subcutaneous and the sublingual being the most commonly used in practice [69,70,71]. A recently published study directly compared the efficacy of subcutaneous with sublingual and intralymphatic and concluded that subcutaneous and intralymphatic were the most effective routes to improve clinical signs [72]. Allergen-specific immunotherapy is complementary to other treatments, as the efficacy takes time to manifest.

Much attention has also been devoted to the identification of biomarkers [73], although it is not clear at this time if these proposed biomarkers are associated with specific responses to treatment. Cytokines such as TSLP [74], thymus and activation-regulated chemokine (TARC) [75], IL-33 [76], and IL-34 [77] have all been described in recent studies as possible biomarkers, and more studies are necessary to understand how these are relevant to a large population of atopic dogs. As we move toward a more targeted approach for the treatment of canine atopic dermatitis, it is reasonable to believe that more biologics targeting these cytokines will become available for dogs. It is important to emphasize that many of these cytokines are produced by keratinocytes (e.g., TSLP, IL-33) and that keratinocytes have the ability to shape the lymphocytic response toward allergens. Cytokines such as IL-33 and TSLP can promote an allergic/inflammatory response rather than tolerance. Additionally, some mediators released by keratinocytes such as TSLP and periostin [78] are able to directly elicit itch by acting on sensory nerves [79]. This is particularly relevant in chronic disease [80] in which increased density of nerve fibers [81] and enhanced peripheral sensitization play a role and can contribute to a decreased response to antipruritic therapy. Thus, keratinocytes are far from being a physical inert barrier, and they are an integral part of a cross talk with the nervous system and the immune cells.

### 2.8. Bacteria and Atopic Dermatitis

Much progress has been made in our understanding of the microbiome in atopic dogs and the importance of restoring biodiversity. We appreciate the role of the microbiome in modulating immunologic responses in dogs and how a dysbiosis can contribute to the development of allergic and inflammatory diseases [82,83]. Decreased cutaneous biodiversity and predominance of *Staphylococcus* is a feature of atopic flares [84,85]. As the antibiotic resistance of *Staphylococcus* grows and represents a serious challenge for clinicians, we have embraced more topical therapy rather than broad spectrum antibiotics and are acutely aware of how important it is to encourage biodiversity and a healthy sustainable microbiome [86]. Interestingly, in human medicine, topical microbiome transplantation has shown promising results for decreasing the severity of atopic dermatitis and the need for anti-inflammatory therapy [87,88]. In veterinary medicine, several studies have shown a positive effect of probiotic supplementation for modulating immune response in atopic dogs [89] and for decreasing the severity of clinical disease and the need for rescue medications [90].

### 2.9. Take Home Message on Canine Atopic Dermatitis

In summary, it is clear that many different factors play a role in shaping the immune response in canine atopic dermatitis and that keratinocytes and the skin microbiome play a crucial role in modulating the immune response to allergens and modulating inflammation and pruritus. Thus, our management needs to be multimodal with the intent to restore skin barrier and biodiversity while we provide relief from the itch and work toward the long-term re-education of the immune system by using allergen-specific immunotherapy. Each patient will have different thresholds and needs for treatment which may change over the life time of the patient and throughout the course of the year, as flare factors lower the threshold of diseases. Thus, the management of these patients becomes an art of applying our current evidence-based information and tailoring it to the individual needs of our patients. Importantly, we should strive to implement a proactive approach that is suitable for the specific case to minimize the need for rescue medications and the development of secondary infections as part of our efforts to minimize the need for systemic antibiotics.

## 3. Cats

### 3.1. Current Understanding on Pathogenesis

Research on feline atopic syndrome has been lagging compared to that on dogs. Recent review papers have focused on cats and atopic syndrome, summarizing the evidence currently published in cats. These papers have proposed new nomenclature [91] to address the fact that cats have their peculiar manifestations of allergic disease which do not exactly match the human or canine disease. The authors of these papers concluded that there is sufficient evidence to accept that cats have atopic disease, a disease in which IgE has been demonstrated to play a role [92,93,94] and which is amenable to allergen-specific immunotherapy. Manifestations of atopic disease in cats can include skin, respiratory, and gastrointestinal disease, as is the case in people. The authors propose using the term “feline atopic skin syndrome” to designate a complex of pruritic inflammatory skin diseases in cats that have a variety of patterns and that are linked to allergen-specific IgE to environmental allergens [95]. This terminology is intended to replace the term “non-flea-non-food hypersensitivity” which was used in the past.

Very little is known at this time about skin barrier dysfunction in atopic cats. Preliminary studies on skin barrier function in atopic cats with skin disease show that transepidermal water loss may be increased, and hydration may, at least in some sites, be decreased [96]. There is limited evidence of any useful correlation between clinical scoring systems and measurements of hydration [97]. Much work needs to be done to assess skin barrier function in atopic cats and its potential relevance to the pathogenesis of the disease. It could be speculated that the development of indolent ulcers and eosinophilic granulomas in the oral cavity of cats could actually be the result of epicutaneous and oral exposure to the allergen, as cats can be vigorous groomers and allergens could make prolonged contact with the perioral and oral mucosa. Currently, there is no study that has documented the development of such lesions in an experimental model of allergen challenge. No study has reported on filaggrin and lipid abnormalities in the skin of allergic cats.

Few studies have reported on the lymphocytic populations [98] in the skin of atopic cats, and increased numbers of CD4+ and CD8+ T cells have been described. We know that IL-4 plays a role in allergic cats [99], and we have preliminary evidence that IL-31 may be relevant in allergic cats, as it is in people and dogs. More specifically, circulating IL-31 levels in cats with a presumptive diagnosis of allergic dermatitis have been reported to be significantly higher than those of normal cats [100]. The authors reported that the mean circulating IL-31 level was 8798 fg/mL for cats with allergic dermatitis compared to 205 fg/mL in age-matched controls. As eosinophils are susceptible to the effects of IL-31 [101], IL-31 can represent an appealing target for the treatment of eosinophilic diseases in cats.

### 3.2. Clinical Disease and How to Make A Diagnosis

**Figure 3:**
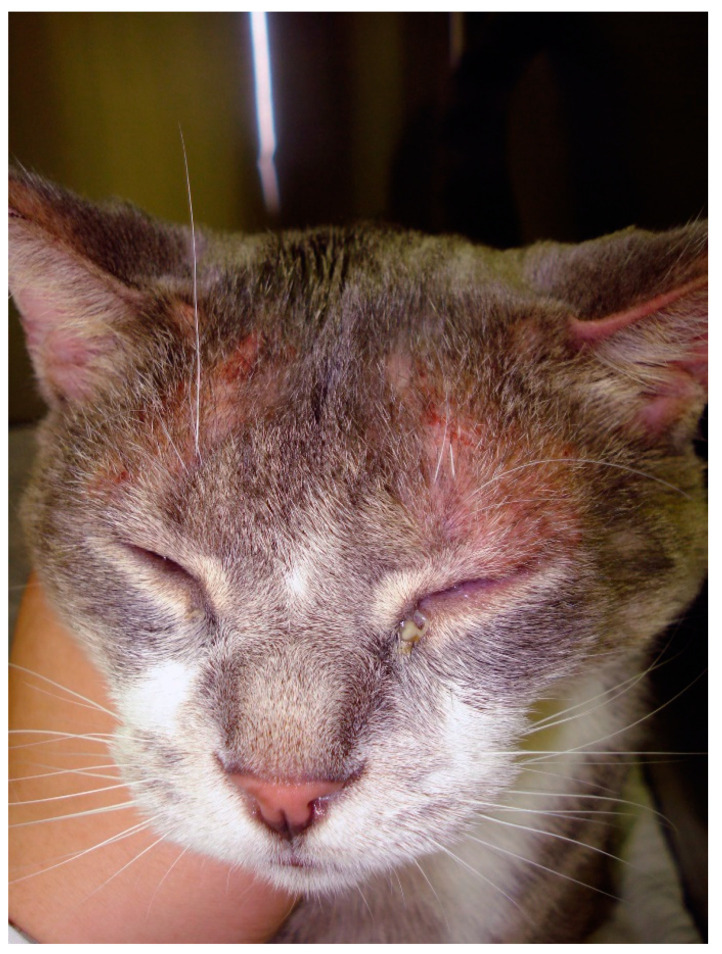
Severe pruritis and facial dermatitis in cat diagnosed with feline atopic skin syndrome. As facial pruritus can be triggered by many diseases, it is important to rule out mites, dermatophytes, and fleas before considering feline atopic skin syndrome as a clinical diagnosis.

The cutaneous manifestations of feline atopic skin syndrome are not the same as those in atopic dermatitis in people and dogs. Some cats can develop facial dermatitis (Figure 3) and pruritus, as in atopic people and dogs, but other feline manifestations of environmental allergies are peculiar to cats (e.g., indolent ulcer, eosinophilic plaque).

Of critical importance is to point out that the clinical manifestations are not pathognomonic for a specific trigger [102,103]. Thus, clinicians cannot make conclusions about the trigger by looking at the clinical presentation of the patient: they need to rule out insects and foods as triggers based on the seasonality and history of the patient and consider the diagnosis of feline atopic skin syndrome (also known as dermatitis linked to environmental triggers) as a diagnosis of exclusion, just as it is in dogs. This requires appropriate flea control in areas where insects are prevalent and an appropriate food trial in patients that are nonseasonal. It is important to point out that although various tests (e.g., salivary test [104], patch test [105]) have been advocated for the diagnosis of food allergy, food trials with a novel source of protein or using a hydrolyzed diet are still the common approach in clinics. The choice of novel protein versus hydrolyzed diet depends on the patient, dietary history, and availability of diets.

Allergy testing (either by use of serology testing or intradermal skin test) can be used to select environmental allergens to include for allergen-specific immunotherapy but not for the purpose of making a diagnosis of feline atopic skin syndrome [106,107,108]. Intradermal skin testing can be technically challenging in cats [109], and serology is frequently used in its place. Cats, similar to people, also have of IgE for cross-reactive carbohydrate determinants which can be responsible for false positive results on serology. The blocking of these IgEs has been reported to improve the accuracy of serology testing [110].

### 3.3. Treatments Available for Atopic Cats

Glucocorticoids and cyclosporine are treatments for which there is the most evidence of efficacy [111]. As part of the multimodal approach, antihistamines and essential fatty acids can be added to the regimen, provide relief in cats with milder disease, and minimize the need for broader spectrum anti-inflammatory therapies [112]. Although oclacitinib is not labeled for cats, some studies have reported on its use in cats with allergic skin disease. One study reporting on the pharmacokinetics of oclacitinib in cats [113] demonstrated that the absorption is variable among individuals and that possibly larger doses may be needed in feline patients compared to dogs. The authors also pointed out that shorter dosing intervals would be recommended in cats to achieve similar blood concentrations to those in dogs. The clinical response to oclacitinib is variable [114] and has overall reported it to be less effective than methylprednisolone [115].

For young atopic cats with long allergy seasons, it is always beneficial to attempt allergen-specific immunotherapy. Thus, identification of environmental allergens that may play a role in that specific patient is important and is done to correlate the results of the allergy testing with the seasonality and environmental exposure of the patient. The formulation of a custom-made vaccine is intended to decrease the dependence on rescue medications. Interestingly, more studies have been published on allergen-specific immunotherapy for feline asthma than for feline atopic skin syndrome. The subcutaneous route has been found to be the most reliable route in feline asthma [116]. Both serology and skin testing were found to be useful for the selection of allergens used for immunotherapy in cats with respiratory disease [117].

There are few studies on allergen-specific immunotherapy in cats with skin disease. A recently published study reported on the efficacy of sublingual immunotherapy in atopic cats sensitized to dust and storage mites [118]. In this prospective open label study, immunotherapy was given for 12 months and monitoring of IgE and IgG was done at various intervals. The authors concluded that a significant decrease of the severity of dermatitis and pruritus was observed at the end of the study, and a decrease of IgE occurred after 9 months, while IgG did not change throughout the study. The treatment was well tolerated and can be considered for cats that do not do well with injections.

A monoclonal antibody against feline IL-31 with the ability to block the binding of this cytokine to its receptor has been described [119], suggesting a promising future biologic for cats.

### 3.4. Take Home Message on Feline Atopic Skin Syndrome

In summary, although we have much work ahead to better understand the pathogenesis of feline atopic skin syndrome, we have some preliminary evidence that similarities may exist in the immune dysregulation between cats and dogs and that IL-31 may be a good target for cats as well. Cats are very much in need of treatments to improve their quality of life, and identification of key cytokines could prove to be of tremendous benefit.

## 4. Horses

### 4.1. Our Understanding on the Pathogenesis in Horses

Horses also develop atopic disease, which can manifest as respiratory or cutaneous disease [120]. The respiratory disease has been recognized to be similar to human asthma [121]. The association between allergen-specific IgE and the presence of dermatitis has been reported in several studies [122,123], and a positive response to allergen-specific immunotherapy in atopic horses has been documented in the literature [124]. Similar to other species, IgE levels are influenced by genetic factors in horses [125].

Our understanding of atopic dermatitis in horses is very limited. It is commonly accepted that this disease is the result of genetic and environmental factors, and it is frequent to see horses that were raised in colder climates manifest disease only later in life when moved to a warmer climate with more insect and environmental pressure. Many of these atopic horses are polysensitized and have hypersensitivity to both insects and various pollens [126]. Very little is known about skin barrier and atopic disease in horses. One study in atopic horses showed ultrastructural abnormalities on electron microscopy when compared to normal horses [127] but it is unclear if this is the result of inflammation or may be suggestive of some primary impairment of the skin which could facilitate the epicutaneous absorption of the allergen and increased risk for allergic sensitization. A recently published study on horses with insect hypersensitivity compared the transcriptome in the epidermis of allergic horses with that of normal horses [128] and suggested skin impairment in insect allergic horses. It is possible that some of these allergic horses were also atopic. Clearly, more work is needed before any conclusion can be made about the existence of a primary skin impairment and what the pathogenetic relevance could be for the equine disease.

### 4.2. Clinical Signs and How to Make A Diagnosis

**Figure 4:**
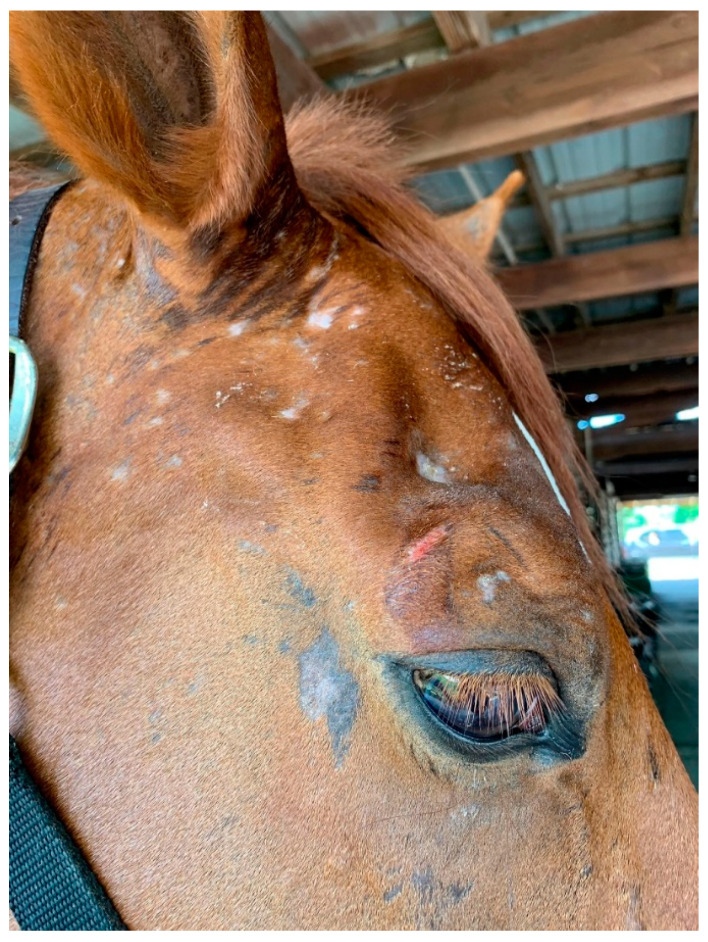
Horse diagnosed with environmental and insect allergies. The pruritus in this patient was intense and led to significant self-trauma.

Atopic dermatitis in horses presents as a relapsing, pruritic inflammatory disease that typically affects the face (Figure 4), ears, and glabrous areas. Some horses may have a history of heaves as well. Many atopic horses are also insect allergic. These patients have clinical signs that are a combination of these allergies: areas like the withers, mane, and tail can all be affected by pruritus. The combination of allergies is an important concept for the control of flares. As it is in small animals where the control of flea exposure is critical to ensure the success of treatment for atopic dermatitis, the prevention of insect bites is important in atopic horses. Controlling all triggers of pruritus is crucial to bring patients below a clinical threshold of pruritus and increase the success of therapy.

As is in other species, the diagnosis of atopic dermatitis is a clinical diagnosis based on suggestive history, compatible clinical signs, and exclusion of other pruritic diseases. This is an important concept as some positive results on both skin testing and serology testing can be seen in normal horses, although in lower numbers when compared to atopics [121,122,129,130]. Nevertheless, allergy testing cannot be used to make a diagnosis of atopic dermatitis [131] similar to other species.

Older studies reported a poor correlation between serology and skin test [132], while newer studies have reported a very good correlation [123]. This may be the result of the more accurate serology testing that is currently available. As we improve our understanding of how to interpret serology testing, we have learned that horses, similar to other species, also have IgEs against cross reactive carbohydrate determinants [133]. The inhibition of these IgEs greatly improves the accuracy of the serology testing.

Treatment of atopic horses primarily still involves the use of glucocorticoids and antihistamines, but no controlled studies have been done to evaluate the efficacy of these treatments in a controlled fashion. The reports of these treatments are retrospective and uncontrolled studies where owners report on the beneficial effects of these strategies [134]. The same limitations hold for reports investigating allergen-specific immunotherapy, which is recommended for atopic horses with a long allergy season [123,135]. The reported success rate of allergen-specific immunotherapy in atopic horses ranges from 64% [133] to 84% [136] depending on the study, and the bulk of the improvement is typically visible after the first year [123]. Some horses can be maintained with allergen-specific immunotherapy alone, while others still require other medications, although the amounts of medications necessary to make them comfortable may be decreased. Importantly, the success of immunotherapy was not reported to be different based on which allergy testing was used to select the allergens [135].

IL-31 has been shown to be a mediator of pruritus in horses, as the injection of IL-31 protein recombinant was able to trigger intense pruritus at the site of injection in normal horses [137]. Thus, IL-31 can be a suitable target for the treatment of pruritus as a preliminary study in insect allergic horses has shown. In this study, allergic horses were immunized against IL-31 and showed a reduction in the severity of clinical scores when compared to a placebo and to their previous season [138]. The authors propose that immunization against cytokines may be a more cost-effective strategy and would also have the benefit of inducing a polyclonal response rather than relying on the administration of an equinized monoclonal antibody in the form of a biologic. The long-term potentially unwanted consequences of immunizing horses against their own cytokines needs further consideration, although other studies targeting vaccination against IL-5 in insect allergic horses seem to support safety even when horses received booster vaccinations for 2 years [139,140].

These strategies are still experimental and allergic horses still suffer from a paucity of treatment options. As in other species, controlling flare factors is of crucial importance. Since many atopic horses are also insect allergic, prevention of insect bites is very important to bring the patient below a threshold of clinical signs. Consistent use of effective repellents is key in geographical areas with a high insect burden. Controlling secondary bacterial infection is also very important to decrease the level of pruritus and prevent self-trauma.

With the goal of providing more options for allergic horses, oclacitinib has also been tested. A dose of 0.25 mg/kg once daily was reported to result in a decrease in severity of signs compared to a placebo starting after 5 days of treatment [141]. No direct comparison between the effect of oclacitinib and glucocorticoids has been published. The once daily dose was used based on information on the pharmacokinetics of this drug in horses [142], which shows a longer half-life compared to dogs.

In summary, our understanding of atopic dermatitis in horses is rudimentary, and much work needs to be done to understand the role of the skin barrier and immune dysregulation in horses, and how this relates to other species. Based on what we know, it is reasonable to hypothesize that skin barrier impairment may exist in horses. Identification of specific targets to decrease the use of glucocorticoids would be of immense benefit. IL-31 appears to be a suitable target.

### 4.3. Dermatitis Linked to Environmental Allergies in Other Animals

**Figure 5:**
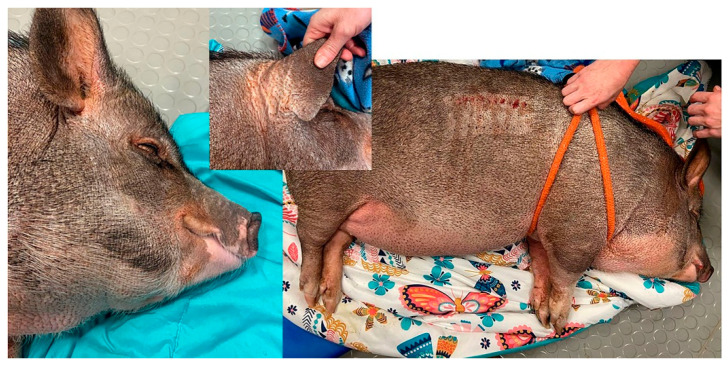
Pot belly pig presenting for pruritic dermatitis of face (periocular and perioral), ventral abdomen, and legs linked to environmental allergies and responsive to allergen specific immunotherapy.

Environmental allergy has been diagnosed in other species ranging from cows to pigs (Figure 5), and even in wildlife species. The author has skin tested and successfully performed immunotherapy in pigs (subcutaneous) and bats (oral) and is aware of oral immunotherapy done in bears. While mice have been used traditionally as a model for atopic dermatitis in people, this species does not spontaneously develop atopic dermatitis. Most rodent models involve some mutation which leads to the development of itch or inflammation, but the dermatitis typically does not mimic the complexity of disease seen in domestic animals.

## 5. Conclusions

Atopic dermatitis affects animals in a similar fashion to the human disease. For animals such as dogs that have embraced lifestyle changes similar to people (e.g., increased exposure to clean, indoor environments and increased consumption of processed foods), these changes have increased the risk of development of allergic disease. Whether increased allergies also are developing in horses and cats due to the fact that animals are dewormed more frequently and therefore have less exposure to parasites than they did in the past remains to be established. Our approach to atopic dermatitis has changed over time to become more holistic and more focused on restoring rather than suppressing the immune system. Sustainability and safety are key for long-term management. Regardless of the species, allergen-specific immunotherapy remains the most desirable long-term option with the intent of re-educating the immune system and decreasing the need of rescue medications. Of all cytokines, IL-31 seems to be a key player across species and the future target of treatment for cats and horses.
